# A Pediatric Case of Middle Cerebral Artery Dissection With Delayed Morphological Changes

**DOI:** 10.7759/cureus.60625

**Published:** 2024-05-19

**Authors:** Noriaki Tanabe, Terushige Toyooka, Arumu Endo, Kojiro Wada

**Affiliations:** 1 Department of Neurosurgery, National Defense Medical College, Tokorozawa, JPN

**Keywords:** computed tomography perfusion, pediatric stroke, magnetic resonance imaging, intracranial artery dissection, middle cerebral artery dissection

## Abstract

Middle cerebral artery dissection (MCAD) is a rare condition with no consensus on its treatment strategy and prognosis. This report describes a case of MCAD with perforating artery infarction in which radiographic findings progressed despite a lack of symptoms following maintenance infusion without antithrombotic therapy. A five-year-old boy presented to our hospital with right hemiplegia. Magnetic resonance imaging revealed diffusion restriction in the left basal ganglia. Additionally, magnetic resonance angiography (MRA) revealed irregular walls in the horizontal portion of the left middle cerebral artery. MRA performed three months after admission revealed progressive stenosis but no new ischemic lesions. MCAD can be associated with long-term morphological changes in the vessel walls. Intracranial artery dissection (IAD) in pediatric patients often presents without headache or neck pain, and serial imaging helps monitor disease progression. In conclusion, the morphology of the vessels can change over several months. Especially in pediatric patients, IAD often presents without headache or neck pain, and serial imaging evaluations help monitor disease progression.

## Introduction

Middle cerebral artery dissection (MCAD) is typically defined as a tear within the vessel wall of the middle cerebral artery (MCA), which can cause cerebral infarction and subarachnoid hemorrhage [1]. MCAD accounts for approximately 4.1% of all intracranial artery dissections and is more frequently reported in Asian countries [1,2]. MCAD is classified into two major types: traumatic and spontaneous. Traumatic MCAD can occur after a minor head trauma [1].

Hemorrhagic cases of MCAD are typically managed surgically [1,3]. However, a treatment strategy for cases of MCAD with ischemia and headache is yet to be established [1]. According to previous reports, cervical artery dissection (CAD) typically does not progress beyond three months [4]. Observational studies have documented cases of intracranial artery dissection that demonstrated ischemia after the onset [5]. However, the evidence for the prognosis of intracranial artery dissection (IAD) is yet to be established.

Here, we report a delayed morphological progression of ischemic MCAD caused by slight head trauma and demonstrate the diagnostic difficulties in pediatric cases.

## Case presentation

A five-year-old boy was examined at the clinic after his mother noticed right hemiplegia upon waking. He was transferred to our hospital with a suspected central nervous system disease.

On the day before symptom onset, the patient hit his head on a wall while rolling forward. He did not experience any neurological deficits and stayed home. On arrival, the patient was conscious; however, he had right hemiplegia, a decline of temperature and pain sensation, slight numbness in the right extremity, and dysphagia.

**Figure 1:**
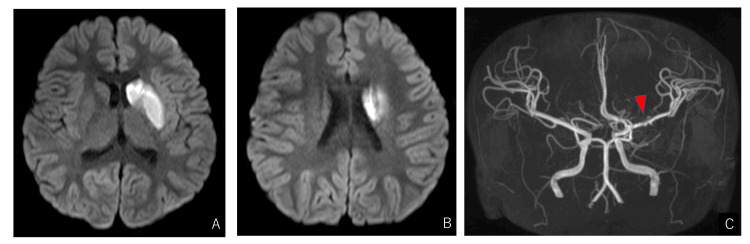
Magnetic resonance imaging at admission. Magnetic resonance (MR) imaging demonstrated diffusion restriction in the left basal ganglia (A, B). MR angiogram revealed irregular morphology in the horizontal portion of the left middle cerebral artery (arrow) (C).

Magnetic resonance (MR) scans taken upon arrival demonstrated diffusion restriction in the left basal ganglia, and MR angiography (MRA) revealed an irregular vessel wall in the horizontal portion of the left MCA (Figure 1).

**Figure 2:**
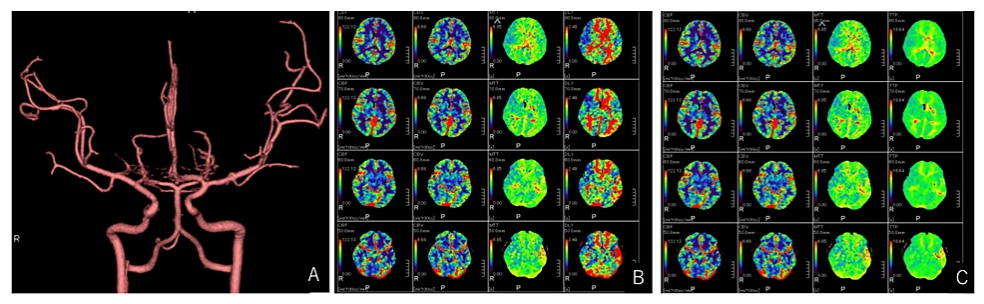
Computed tomography (CT) angiography and perfusion imaging after admission. CT angiography on the day after admission displayed no progression of arterial dissection (A). CT perfusion on the same day revealed decreased cerebral blood flow and cerebral blood volume at the infarct site. Still, cerebral blood flow in the peripheral portion of the middle cerebral artery was preserved (B, C).

MCAD was suspected, and the patient was treated with maintenance infusion without antithrombotic therapy. Computed tomography (CT) angiography performed the day after admission showed no obvious progression of the dissection (Figure 2A). CT perfusion revealed decreased cerebral blood flow (CBF), consistent with the site of the known cerebral infarction, but no evidence of prolonged blood flow in other areas (Figures 2B, 2C).

**Figure 3:**
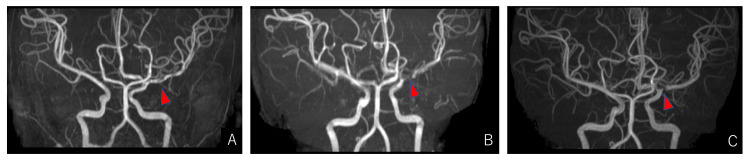
Magnetic resonance angiography of morphological changes of left middle cerebral artery. On day 15, the extent of vessel stenosis progressed and demonstrated a "pearl-and-string sign" (arrow) (A). Further progression of stenosis was observed three months after onset (arrow) (B). No progression was observed three years and four months after onset (arrow) (C).

No deterioration in neurological deficits was observed after admission. On day 15, after admission, the patient presented with a mild headache and vomiting. MR imaging (MRI) demonstrated edematous changes and swelling at the cerebral infarction site. MRA exhibited progressive wall irregularity of the MCA with pearl-and-string signs (Figure 3A). We took a watch-and-wait approach with the patient as the extent of cerebral ischemia did not increase. After two months of physical therapy, MRA performed three months after the onset demonstrated further progression of the stenosis, but no new cerebral infarction was observed (Figure 3B).

The patient was continuously followed up, but no new symptoms were observed. MRI three years and four months after onset revealed no novel cerebral infarction, with an excellent peripheral projection of the MCA (Figure 3C).

## Discussion

In this case, we highlighted two key points: first, MCAD has the potential for long-term disease progression and requires long-term follow-up. Second, pain and symptoms may not appear in pediatric patients, and continuous imaging helps identify lesion progression, even in the diagnosis of IAD.

Radiographical changes of MCAD

Extracranial artery dissection has a high recanalization rate and rarely exhibits long-term progression [4]. However, few studies have investigated the long-term prognosis of IAD, including MCAD [2]. In the present case, MRA performed three months after admission revealed progression of the left MCA lesion. However, owing to the intermittent timing of the MRI evaluation, it was difficult to determine whether the dissection was persistently progressive or recanalized after achieving hemostasis. Current guidelines recommend the administration of antiplatelet agents in patients with IAD [6]. In the present case, antiplatelet agents were not administered; however, delayed changes in the appearance of the vessels occurred. Although there is no evidence that administering antiplatelet agents increases the risk of arterial dissection progression, long-term evaluation of vascular morphology should continue.

Symptoms of MCAD

The symptoms of childhood stroke are nonspecific, and diagnosis is often delayed, requiring significant time for appropriate treatment. This is attributed to symptoms that frequently include somnolence, irritability, anorexia, vomiting, and, rarely, focal symptoms [7,8]. It is reported that 7.5% of pediatric patients with arterial ischemic stroke were diagnosed with craniocervical artery dissection, including MCAD [9]. In addition, approximately half of the childhood patients with IAD present with headaches and cervical symptoms, whereas the other half do not exhibit headache or neck pain [10]. In the present case, the patient presented with right hemiplegia but did not have a headache or neck pain. Diagnosing MCAD would have been challenging without an MRI at the time of presentation to identify morphological changes in the horizontal portion of the MCA.

Diagnoses of MCAD

The diagnostic criteria for IAD require meeting at least one of the following conditions: (i) a stenosis or occlusion of an intracranial artery secondarily developing towards a fusiform or irregular aneurysmal dilation at a non-branching site; (ii) an intramural hematoma, intimal ﬂap, or double lumen; and (iii) pathological conﬁrmation of IAD [6]. In this case, MRA demonstrated pearl-and-string signs, but evaluation of the intramural hematoma with non-contrast T1-weighted thin-slice imaging was complex because of motion artifacts. The difficulty in the detailed assessment of radiograms makes diagnosing MCAD difficult in pediatric patients. Repeated imaging is necessary if the diagnosis after a single evaluation is challenging.

## Conclusions

Middle cerebral artery dissection can occur in young patients, and a recommended follow-up duration has not yet been established. In this case, the morphology of the vessels changed over several months. In pediatric patients, intracranial artery dissection often presents without headache or neck pain, and serial imaging evaluations help monitor disease progression.

## References

[1] Asaithambi G, Saravanapavan P, Rastogi V (2014). Isolated middle cerebral artery dissection: a systematic review. Int J Emerg Med.

[2] Mizutani T (2011). Natural course of intracranial arterial dissections. J Neurosurg.

[3] Hashimoto H, Iida J, Shin Y, Hironaka Y, Sakaki T (1999). Subarachnoid hemorrhage from intracranial dissecting aneurysms of the anterior circulation. Two case reports. Neurol Med Chir (Tokyo).

[4] Engelter ST, Traenka C, Lyrer P (2017). Dissection of cervical and cerebral arteries. Curr Neurol Neurosci Rep.

[5] Nakagawa K, Touho H, Morisako T (2000). Long-term follow-up study of unruptured vertebral artery dissection: clinical outcomes and serial angiographic findings. J Neurosurg.

[6] Debette S, Mazighi M, Bijlenga P (2021). ESO guideline for the management of extracranial and intracranial artery dissection. Eur Stroke J.

[7] Cerrato P, Grasso M, Imperiale D (2004). Stroke in young patients: etiopathogenesis and risk factors in different age classes. Cerebrovasc Dis.

[8] Tsze DS, Valente JH (2011). Pediatric stroke: a review. Emerg Med Int.

[9] Rafay MF, Armstrong D, Deveber G, Domi T, Chan A, MacGregor DL (2006). Craniocervical arterial dissection in children: clinical and radiographic presentation and outcome. J Child Neurol.

[10] Amlie-Lefond C, Sébire G, Fullerton HJ (2008). Recent developments in childhood arterial ischaemic stroke. Lancet Neurol.

